# Machine learning–based identification and ranking of risk factors for lumbar paraspinal muscle atrophy

**DOI:** 10.1007/s00402-026-06256-w

**Published:** 2026-03-28

**Authors:** Lukas Schönnagel, Tom Folkerts, Ali Guven, Erika Chiapparelli, Jiaqi Zhu, Gaston Camino-Willhuber, Thomas Caffard, Artine Arzani, Paul Köhli, Marco D. Burkhard, Jennifer Shue, Andrew A. Sama, Federico P. Girardi, Frank P. Cammisa, Alexander P. Hughes

**Affiliations:** 1 Center for Musculoskeletal Surgery, Charité - Universitätsmedizin Berlin, corporate member of Freie Universität Berlin and Humboldt-Universität zu Berlin, Berlin, Germany; 2 Departement of Orthopaedic Surgery, Hospital for Special Surgery, New York, USA; 3 Biostatistics Core, Hospital for Special Surgery, New York, USA; 4 Klinik für Orthopädie, University Hospital Ulm, Ulm, Germany

**Keywords:** Lumbar spine, Muscle atrophy, Paraspinal muscles, Machine learning, Fatty infiltration

## Abstract

**Background:**

Recent studies highlight the crucial role of the paraspinal musculature (PM), particularly the multifidus (MF), in spinal health and patient outcomes. However, factors associated with PM atrophy and their relative importance remain unclear. To address this gap, we analyzed factors linked to PM atrophy in patients undergoing lumbar fusion using machine learning, aiming to clarify the multifactorial mechanisms underlying this condition.

**Methods:**

Fatty infiltration (FI) of the lumbar MF was measured as a proxy for muscular atrophy in patients undergoing lumbar spinal fusion. Two machine learning models, logistic regression and extreme gradient boosting (XGBoost), were trained to predict severe FI (> 50%) of the MF. Model performance was evaluated on unseen test data using receiver operating characteristic (ROC) analysis and Brier score, and predictor importance was assessed via SHAP (SHapley Additive exPlanations).

**Results:**

The study included 316 patients, primarily treated due to lumbar spinal stenosis. Both machine learning models effectively predicted severe MF atrophy, with an area under the curve (AUC) of 0.83 (95% CI 0.74–0.83) for the logistic regression and 0.88 (95% CI 0.81–0.88) for the XGBoost. In the logistic regression model, only sex, age, and facet joint degeneration were significant predictors. The XGBoost model identified the same top three variables, while the lumbar endplate score and bone mineral density ranked higher than in logistic regression.

**Conclusion:**

This study introduces a novel framework for analyzing factors influencing PM atrophy, highlighting the intricate interplay between demographic variables like age and sex and facet joint degeneration. By applying modern machine learning techniques, we improved predictive accuracy and identified endplate and bone changes as strongly associated factors, offering valuable insights into the mechanisms shaping muscle health in lumbar conditions.

## Introduction

The integrity of the spinal column relies on the surrounding paraspinal musculature (PM) [1, 2]. Biomechanical and clinical studies demonstrate that these muscles play a pivotal role in intersegmental stability and coronal and sagittal alignment of the spine. (3–5) The posterior PM plays an especially crucial role in load distribution by transforming bending forces due to asymmetric loading into compressive forces, reducing stress on the intervertebral disc and other passive stabilization structures like the facet joints and spinal ligaments [3]. Thus, impairment of the PM is implicated in increased intradiscal forces and increased occurrence and severity of lower back pain (LBP) [4, 5]. The clinical relevance of PM atrophy, especially the multifidus muscle (MF), has also been shown in recent reviews that demonstrated worse patient-reported outcomes after spinal surgery and increased post-operative complications in patients with paraspinal muscle atrophy [6, 7]. 

Historically, numerous studies could identify individual factors that are associated with the PM atrophy. These include nerve compression [8], as well as local inflammation [9], and atherogenic muscle inhibition due to intervertebral disc degeneration and facet joint arthropathy [10, 11]. Similarly, demographic factors like age [12], sex [13], vascular changes [14], and collagen degradation [15] have been reported to negatively affect skeletal musculature. Still, the comparative importance of these factors is unclear. The interaction of the individual factors and a potential cyclic relationship between each factor and the PM themselves further complicates this ambiguity. Moreover, despite known associations of these factors with muscle deterioration at a population level, no predictive models for individual patient assessment exist. Given the increasingly recognized role of the PM in spinal health and patient outcomes, we believe a more comprehensive and comparative analysis is needed.

The objective of this study was to assess and rank the association of established and novel factors associated with atrophy of the MF. For this, we analyzed a population of patients undergoing spinal surgery due to degenerative pathologies and developed two machine-learning models to predict severe PM atrophy in individual patients. Second, we analyzed the feature importance of these models to rank the particular risk factors. The first machine learning model utilizes a classical approach, using a linear logistic regression model. As the interaction may not be linear, we also employed a more advanced machine learning technique, extreme gradient boosting (XGBoost) [16], which is increasingly used in clinical studies [17–19]. By integrating a wide range of structural and systemic factors into a single predictive model, this work aims to provide a more comprehensive approach to characterizing and ranking key associated factors of paraspinal muscle degeneration.

## Methods

### Patient population

Approval of the hospital’s institutional review board (IRB) was granted before data collection (IRB#2019–2137). We conducted a retrospective review of the medical records of patients who underwent lumbar spinal surgery due to degenerative conditions. Exclusion criteria included a history of lumbar spinal fusion and the absence of adequate magnetic resonance imaging (MRI) data. Surgeries took place from January 2016 to July 2023. This study aligns with the Strengthening the Reporting of Observational Studies in Epidemiology (STROBE) guidelines.

### Measurement of the paraspinal muscles

**Fig. 1:**
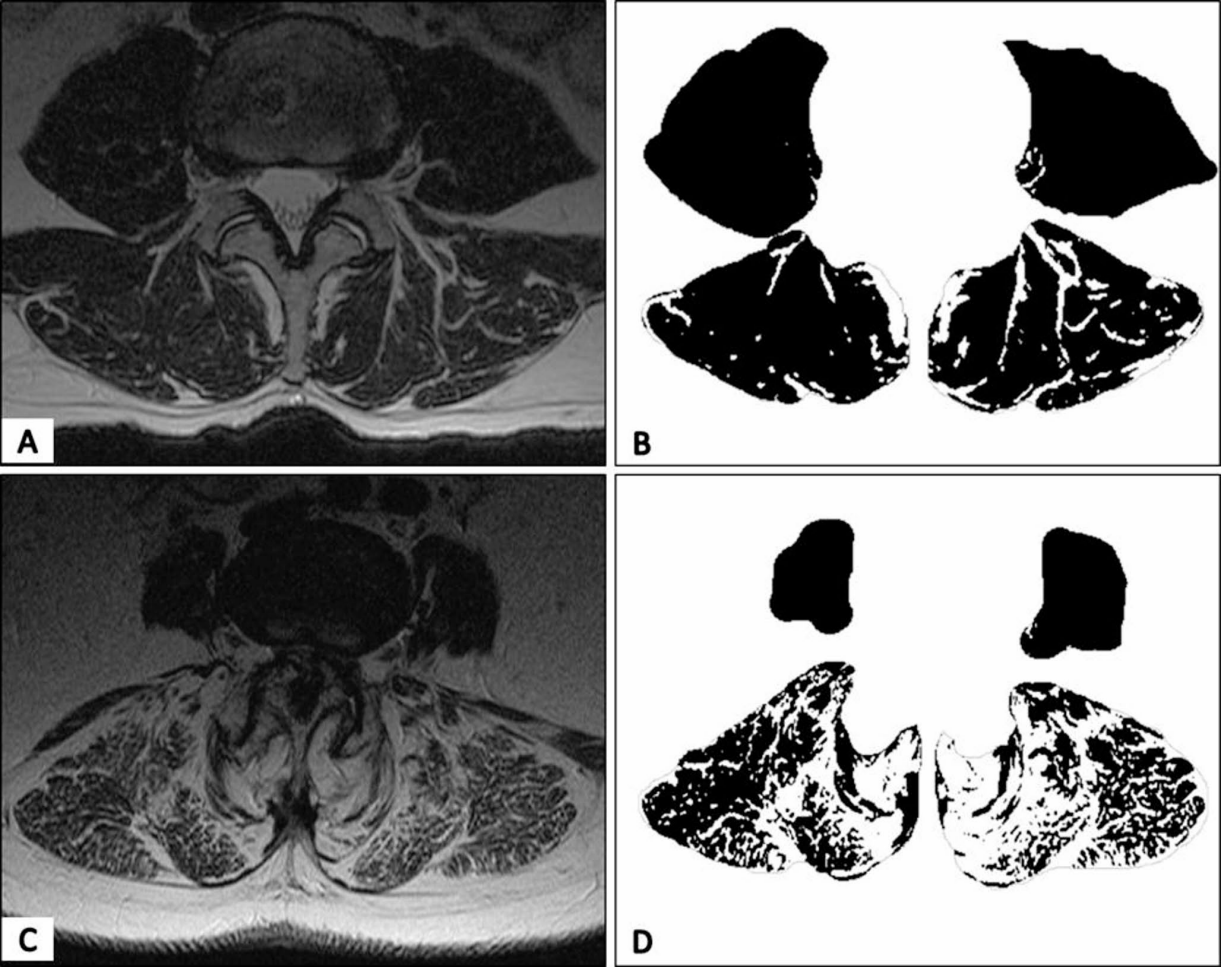
Muscle Measurement

We assessed MF atrophy by measuring the fatty infiltration (FI) in T2 weighted axial MRI sequences. For this, we used specialized software (ITK SNAP version 3.8.0) to segment the MF at the level of the upper endplate of L4, which has been used in multiple previous studies. The muscle’s total cross-sectional area (CSA) was determined using custom software written in MATLAB (MathWorks, Inc., 2022) [20, 21]. To determine the threshold between fat and muscle tissue, we first used the widely used intermeans thresholding algorithm and then determined the mean and standard deviation of pixels below this threshold to calculate the final threshold, used in previous studies [20, 21] A depiction of this process is in Fig. 1. The percentage of FI for the posterior PM was calculated with the following formula:

$$ {\mathrm{FI}}\, \% \,{\text{ = }}\,\frac{{{\text{Fat Area}}}}{{{\mathrm{CSA}}}}\,*\,100 $$

Figure 1 depicts the methodology for evaluating fatty infiltration within the paraspinal muscles. Panels A and C present an MRI cross-section at the L4 upper endplate level. Initially, the paraspinal muscle is segmented. Subsequently, a modified intermeans algorithm is applied to distinguish between muscle and fatty tissues. Panels B and D illustrate the completed segmentation and thresholding, with black indicating muscle tissue and white denoting adipose tissue.

### Selection of predictor variables

In the initial selection of variables, we reviewed the existing literature and used clinical expertise to identify relevant domains of predictors. Demographic variables included age, sex, BMI, and smoking. Comorbidities included arterial hypertension, diabetes mellitus, and aortic abdominal calcification [14]. Radiologic parameters included established measures of spinal degeneration, including disc degeneration (Pfirrmann) [22], degeneration of the vertebral endplate [23], the severity of spinal stenosis measured according to the Schizas classification [24], and severity of foraminal stenosis measured according to Lee et al. [25] Disc-, endplate-, and facet joint degeneration were each aggregated into a lumbar score, combining the individual score at each lumbar level. According to previous research, spinal stenosis above the measured level of musculature (upper endplate of L4) and foraminal stenosis at the L3/4 were evaluated as these were reported to have the largest effect on multifidus atrophy [26, 27]. Additionally, bone mineral density (BMD) was assessed using Quantitative Computed Tomography (QCT). Following the literature, the mean QCT of L1 and L2 was used [28]. Systemic collagen degradation was assessed by measuring the echogenicity of the lower third of the dermis, which is reported to be a proxy for advanced glycation end products in previous research [15, 29]. Systemic weakness was assessed with the modified 5-item Frailty score, often used in spinal research [30, 31]. All parameters were assessed preoperatively.

### Development of a predictive machine learning model

Based on thresholds used by previous studies, the outcome of the machine learning models was atrophy of the MF exceeding 50% FI [6]. Two models were developed and compared: linear logistic regression and the novel Xtreme Gradient Boosting (XGBoost). Linear logistic regression is a statistical method for modeling the relationship between one or more independent variables and a binary dependent variable [16]. XGBoost is an optimized gradient-boosting machine-learning library. It utilizes an ensemble of decision trees, learning from previous trees’ errors to iteratively improve predictions. A random forest algorithm performed the initial ranking of importance. For the number of predictor variables in the final models, we adhered to the ‘10 per event’ rule to mitigate the risks of overfitting and miscalibration [32]. 

Missing values of variables with less than 15% missing values were imputed using the “missForest” library. This uses a random forest machine learning algorithm to predict missing values. The data was then partitioned into a training and validation set, with a 70:30 split. The training set was used to develop machine learning models. The model’s performance was subsequently evaluated in the unseen validation dataset, an important step in preventing the overfitting of the model. The primary outcome of this analysis is the Area under the Curve (AUC) [22]. An AUC of 0.7 to 0.8 was considered fair, 0.8 to 0.9 good, and ≥ 0.9 excellent [23]. Additionally, the models’ accuracy, sensitivity, and specificity were reported. The calibration of the model was assessed using the Brier score. The Brier score quantifies how well the model’s predicted probabilities match the outcomes. It calculates the mean squared difference between the predicted probability of an event and the actual event occurrence, with lower scores indicating better model prediction accuracy [33]. 

**Fig. 2:**
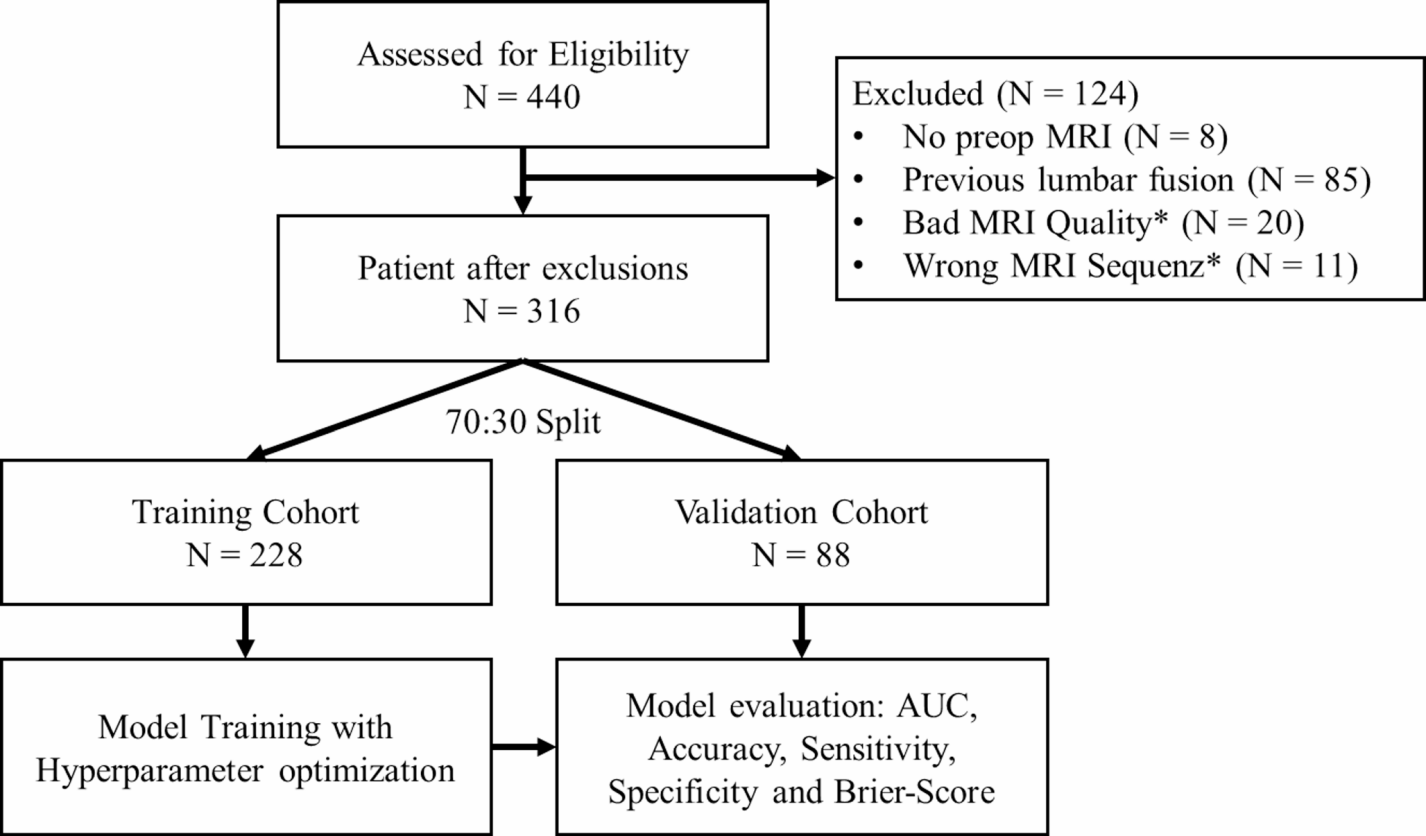
Study flowchart and model development

To evaluate the importance of features in the logistic regression model, we normalized the features using Z-score normalization and ranked the estimates of the individual features [34]. Feature importance in the XGBoost model was evaluated using SHAP (SHapley Additive exPlanations). SHAP values provide a unified measure of feature importance by assigning each feature an important value for a particular prediction based on the average contribution of that feature across all possible combinations of features [35]. The full process is illustrated in Fig. 2. All statistical analyses were conducted utilizing the R programming language within the RStudio environment (Posit PBC, Boston, MA). Statistical significance was determined based on a threshold of *p* < 0.05.

## Results

### Patient characteristics

A total of 316 patients were included in the analysis. Exclusions are listed in Fig. 1. The median age was 63 years (55–71), the median BMI was 28 kg/m^2^ (25–32), and 48.7% were female. 180 (57%) of patients had MF FI greater than 50%. The most common pathology present as part of the operative diagnosis was lumbar spinal stenosis (80.8%), followed by degenerative lumbar spondylolisthesis at L4/5 (55.6%). A concurrence of multiple diagnoses was possible. No feature was missing in more than 15% of patients. The feature with the most missing data was echogenicity of the dermis (10.7%), closely followed by facet joint arthropathy (10.2%). A complete list of patient characteristics selected in the initial random forest model and differences between patients with and without MF atrophy greater than 50% is in Table 1.

After enforcing the exclusion criteria, 316 patients remained eligible for the study. Of these, 70% were randomly assigned to the training cohort, where various machine-learning models were developed and refined. The remaining 30% formed the validation cohort, in which the efficacy of the optimized models was assessed using metrics such as the Area Under the Receiver Operating Characteristic Curve (AUC), Brier Score, and additional performance parameters.

**Table 1:** Demographics

Age	All (*n* = 316)	MF FI < 50% (*n* = 136)	MF FI ≥ 50% (*n* = 180)	*P* – Value
	63 (55–71.2)	56 (48.7–62)	69 (62–74)	< 0.001
BMI	28.3 (25.2–32.5)	27.9 (24.7–31.3)	29.4 (25.8–33.7)	**0.028**
Male	162 (51.3%)	87 (64%)	75 (41.7%)	**< 0.001**
Female	154 (48.7%)	49 (36%)	105 (58.3%)	**< 0.001**
Lunbar spinal stenosis	253 (80.8%)	100 (74.6%)	153 (85.5%)	**0.023**
DLS at L4/L5	174 (55.6%)	58 (43.3%)	116 (64.8%)	**< 0.001**
Degenerative Scoliosis	61 (19.5%)	13 (9.7%)	48 (26.8%)	**< 0.001**
DM (Yes)	33 (10.5%)	10 (7.4%)	23 (12.8%)	0.176
HTA	140 (44.4%)	43 (31.9%)	97 (53.9%)	**< 0.001**
CHF	17 (5.4%)	5 (3.7%)	12 (6.7%)	0.362
Dermal Echogenicity	115.6 (100–129.4)	113.2 (96.6–127.3)	116.4 (101.9–131.7)	0.277
QCT (L1/2 Average)	112.7 (90.4–139.3)	124.7 (105.7–146)	104.5 (83.1–125.6)	**< 0.001**
AAC24	0 (0–4)	0 (0–2)	2 (0–6)	**< 0.001**
Foraminal Stenosis L3/4	2 (1–3)	1 (0–3)	2 (1–4)	**< 0.001**
Lumbar Pfirrmann Grade	18 (16–20)	17 (14–18)	19 (17–21)	**< 0.001**
Lumbar Facet score	14 (9–18)	10 (6–13)	17 (13–21)	**< 0.001**
modified 5 Item Frailty Score	1 (0–1)	0 (0–1)	1 (0–1.5)	**< 0.001**

Participants with greater than 50% fatty infiltration (FI) in the multifidus (MF) were typically older, had a higher BMI, and a greater proportion were female, compared to those with less than 50% MF FI. They also showed more severe spinal degeneration, higher rates of lumbar spinal stenosis and degenerative scoliosis, and were more likely to have hypertension. Bone density was lower in this group, as indicated by QCT scores. Other factors, such as diabetes, heart failure, and degenerative spondylosis, did not differ significantly between the groups. TEPS = total endplate score, BMI = body mass index, QCT = quantitative computed tomography

### Model performance

Both models predicting MF atrophy demonstrated good accuracy in the ROC analyses using the validation dataset. The logistic regression model demonstrated an AUC of 0.81 (95% CI 0.71–0.90), and the XGBoost model had a slightly higher AUC of 0.87 (95% CI 0.79–0.94). The full evaluation of the models is listed in Table 2.

**Table 2:** Model Evaluation

LR	AUC	Accuracy	Sensitivity	Specificity	Brier score
	0.81 (95% CI: 0.71–0.90)	0.70 (95% CI: 0.60–0.80)	0.70	0.82	0.18
XGBoost	0.87 (95% CI 0.79–0.94)	0.81 (95% CI 0.71–0.88)	0.86	0.72	0.14

This table compares the performance of Linear Regression and the XGBoost model in predicting paraspinal muscle atrophy. Key metrics include AUC, Accuracy, Sensitivity, Specificity, and Brier Score. The XGBoost model demonstrates superior performance in most metrics, particularly in AUC and Accuracy, indicating a more accurate prediction of severe atrophy. The lower Brier scores for XGBoost suggest better overall prediction accuracy. LR = Linear Regression, AUC = Area Under the Curve

### Feature importance

The most important feature in the logistic regression model was age, with a normalized odds ratio (OR) of 3.49 (95% CI 2.7–4.2), followed by sex and facet degeneration, with an OR of 3.37 and 2.27, respectively. All these features were independently significant (*p* < 0.01). OR for non-binary parameters were Z-Score normalized. In this normalized estimate, with each standard deviation increase of the predictor (for example, age), the risk of severe MF atrophy increases by the respective OR. For the binary variable sex, the OR means that females were 3.4 times more likely to suffer from severe MF atrophy. The full model with normalized and raw OR is given in Table 3.

**Table 3:** Logistic Regression Model

Sex	Odds-Ratio (Z-Score normalized)	*P*-Value
	3.37 (95% CI 2.91–3.83)	0.000
Age	3.49 (95% CI 2.73–4.24)	**0.001**
Lumbar Facet Score	2.27 (95% CI 1.77–2.77)	**0.001**
Foraminal Stenosis L3/4	1.52 (95% CI 1.07–1.97)	0.066
BMI	1.3 (95% CI 0.89–1.7)	0.208
Lumbar TEPS	1.35 (95% CI 0.84–1.87)	0.246
Lumbar Pfirrmann Grade	0.74 (95% CI 0.12–1.36)	0.345
QCT (L1 + L2)	0.82 (95% CI 0.35–1.29)	0.404
modified 5-item Frailty Score	1.14 (95% CI 0.71–1.56)	0.554
Spinal Stenosis (L1-L3)	0.91 (95% CI 0.52–1.29)	0.618
AAC24-Score	1.08 (95% CI 0.68–1.48)	0.711
Dermis Echogenicity	0.89 (95% CI 0.1–1.67)	0.765

Table 3 shows the feature importance in the logistic regression model, with the z-score normalized odds ratios reflecting the increased risk per standard deviation change in the predictor. The binary variables sex status remained unnormalized. Age, sex, lumbar facet score, and foraminal stenosis at L3/L4 are identified as the most important and independent significant factors. The threshold for statistical significance was set at a p-value of 0.05. TEPS = total endplate score, BMI = Body mass index, QCT = Quantitative Computertomographie.

**Fig. 3:**
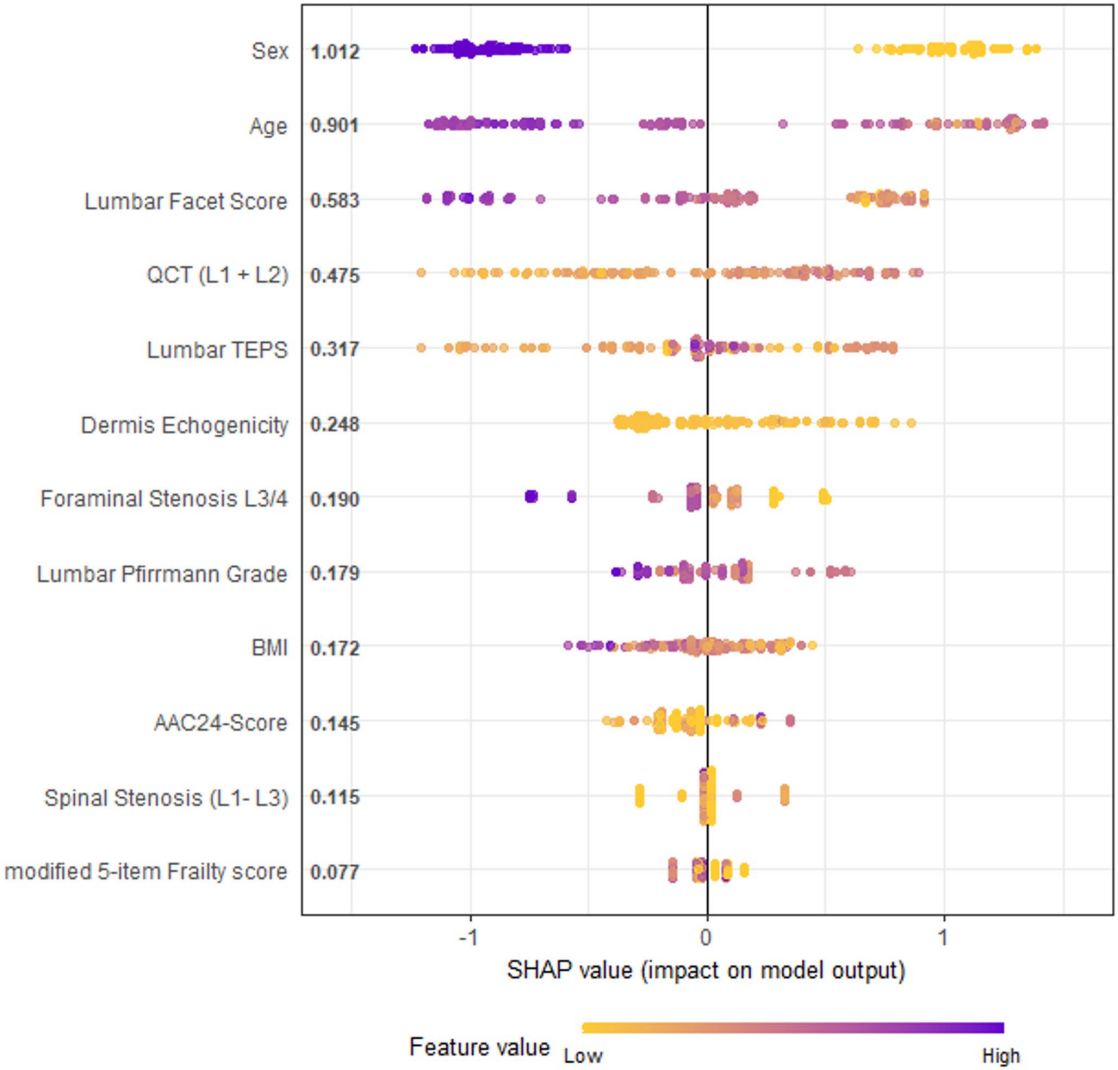
SHAP Importance

In the SHAP feature importance evaluation of the XGB model, sex, age, and facet degeneration also demonstrated the highest importance, with relative SHAP values of 1.012, 0.901, and 0.583 respectively. Compared to the linear regression model, QCT, Endplate Degeneration, and dermal ultrasound values (as a proxy for advanced glycation end products) ranked in the top 6, with values of 0.475, 0.317 and 0.248, respectively. The full list is shown in Fig. 3.

Figure 3 depicts the significance of different predictors in the XGBoost model using SHAP (SHapley Additive exPlanations) values. A negative value on the x-axis correlates with a higher probability of severe multifidus atrophy. For instance, an elevated lumbar facet score, indicated in purple, is strongly linked to an increased risk of significant atrophy, holding a SHAP value of 0.699. This contrasts with sex, which has a SHAP value of 1.203. Consistent with the logistic regression results, sex, age, and lumbar facet score rank as the top three factors. Notably, the model also emphasizes bone density, as measured by quantitative computed tomography (QCT), and the echogenicity of the dermis, positioning them within the five most influential factors.

## Discussion

This study aimed to analyze associated factors of paraspinal musculature atrophy. For this we developed two machine learning models, to predict MF FI above 50% and analyzed the importance of individual features. Both models demonstrated good to excellent predictive ability to predict severe MF atrophy in patients not used for model training, though the XGBoost model performed better than the classical regression model. The strong predictive performance of these models lends credibility to their feature importance rankings, indicating that these rankings may provide meaningful insights into the relative contributions of individual predictors to MF atrophy.

The primary findings of this study indicate that MF atrophy was most strongly associated with age, sex, and facet joint degeneration in both the linear logistic regression and XGBoost models. While these results are already reported in previous literature, our findings extend this knowledge by quantifying their relative contribution and confirming their dominant influence within a multivariable framework. Our model also not only identified risk factors, but was able to predict severe atrophy for individual patients. Notably, in the more accurate XGBoost model, additional factors such as QCT, endplate degeneration, and lower dermis echogenicity ranked among the top six predictors, despite not being significant in the logistic regression model. The XGBoost model’s superior performance highlights the potential of advanced machine learning techniques to better capture complex relationships between factors contributing to paraspinal musculature atrophy. Unlike traditional regression models, XGBoost accounts for intricate interactions and non-linear associations among predictors, offering a more nuanced understanding of their relative importance [36]. 

Our findings align with and expand on previous studies that identified risk factors for skeletal muscle atrophy. Many studies, recently summarized in a review by Wilkinson et al., have identified muscle atrophy as age-dependent, resulting from hormonal changes, reduced physical activity, and nutritional deficiencies. These are estimated to lead to a loss of 1% of muscle mass per year, starting from middle age [12]. Similarly, female sex is associated with higher muscle fat infiltration, partly due to hormonal and societal differences in musculature [13]. Our study corroborates and highlights the strong association with facet joint arthropathy found in previous studies [37], further underscoring the relevance of local degenerative processes in the pathophysiology of muscle deterioration. The current hypothesis is that local inflammation and nerve compression directly lead to muscle atrophy or an atherogenic inhibition of muscular contraction, leading to degenerative changes due to disuse [9, 10]. Conversely, the potential for a reverse causality exists, whereby atrophy of the PM could precipitate spinal instability, subsequently increasing mechanical strain on the facet joints and promoting degeneration. Given the cross-sectional nature of our study, it is beyond the study’s scope to determine the directionality of this relationship.

Moreover, our research suggests a link between advanced glycation endproducts (AGEs) and skeletal muscle atrophy. The echogenicity of the lower dermis has been linked to increases in AGEs of collagen fibers (29), which in turn have been described as a pathomolecular mechanism underlying age-dependent muscle atrophy. (15) The accumulation of AGEs, which we found to be relevant in our more accurate XGBoost model, has been previously associated with general skeletal muscle degeneration, but not with the PM specifically [15]. The importance of the PM has been discussed for multiple decades [1, 38], and supported by biomechanical findings that demonstrate their critical role in intersegmental stability and load distribution [3, 39, 40, 1]. The clinical importance has been demonstrated by an association with other spinal pathologies, such as sagittal malalignment [41] and increased intervertebral disc degeneration [42]. Similarly, recent reviews and studies have identified the atrophy of these muscles as an independent risk factor for worse patient-reported outcomes [6, 43] and an increased risk of complications after surgery [7]. Given the clinical importance of this field of research, this study is a significant step towards a more comprehensive understanding of this interplay and future therapeutic strategies. In addition to physiotherapy, advanced treatment strategies applied in other musculoskeletal conditions, such as biological scaffolding may hold promise for targeted regeneration of paraspinal muscle tissue [44]. Moreover, device-based neuromodulation has emerged as a potential approach to counteract denervation-related atrophy and restore neuromuscular function in affected patients [45]. The primary strength of this study lies in its use of machine learning techniques to comparatively rank factors associated with multifidus atrophy. In contrast to conventional regression-based approaches, this methodology provides a more nuanced understanding of the multifactorial and interdependent mechanisms underlying paraspinal muscle degeneration and may enable individualized risk prediction. By leveraging a large, clinically relevant cohort and integrating both structural and systemic parameters into a unified analytical model, this study offers a refined perspective on the complex contributors to muscle atrophy and strengthens the translational relevance of its findings. Furthermore, the developed framework may provide the basis for future predictive applications designed to identify patients at risk earlier and to guide more personalized preventive or therapeutic interventions.

Despite these strengths, this study has several limitations. Primarily, the dependency on MRI-based features limits the model’s applicability to patients who do not undergo this imaging modality. Future studies should add to this framework with more widely available data including patient activity and exercise habits. Additionally, the cross-sectional design inhibits our understanding of how the identified factors affect paraspinal muscle atrophy over time. The study design also prohibits a clear establishment of causation between individual factors and PM atrophy or if PM atrophy influences this factor. To determine this, interventional and experimental studies are needed. Nonetheless, we believe that our outcomes and techniques serve as a foundation for future research. Finally, concerning external validity, while the study’s findings are robust for its cohort, they do not apply to other populations such as non-surgical patients. Replication and validation in other diverse settings will be essential for ensuring broader applicability.

## Conclusion

This study provides a comprehensive analysis of paraspinal muscle atrophy using both conventional statistical and machine learning approaches. Our findings identify age, sex, and facet joint degeneration as the primary factors associated with multifidus degeneration, while also revealing the contributory roles of endplate changes, bone quality, and systemic connective tissue alterations. While further studies are needed to validate and expand these findings, our results demonstrate the potential of machine learning for individualized risk assessment and targeted prevention of paraspinal muscle degeneration.

## Data Availability

Data availability statementThe datasets generated and analyzed during the current study are available from the corresponding author upon reasonable request.
